# High Occurrence of Pathogenic Free-Living Amoebae in Arid Environments

**DOI:** 10.3390/pathogens15010041

**Published:** 2025-12-30

**Authors:** Patricia Pérez-Pérez, Javier Chao-Pellicer, Rubén L. Rodríguez-Expósito, Marco Peña-Prunell, Angélica Domínguez-de-Barros, Omar García-Pérez, Elizabeth Córdoba-Lanús, María Reyes-Batlle, José E. Piñero, Jacob Lorenzo-Morales

**Affiliations:** 1 Instituto Universitario de Enfermedades Tropicales y Salud Pública de Canarias (IUETSPC), Universidad de La Laguna (ULL), 38206 San Cristóbal de La Laguna, Spain; pperezpe@ull.edu.es (P.P.-P.); jchaopel@ull.edu.es (J.C.-P.); rrodrige@ull.edu.es (R.L.R.-E.); marcodpp@gmail.com (M.P.-P.); adoming@ull.edu.es (A.D.-d.-B.); ogarciap@ull.edu.es (O.G.-P.); acordoba@ull.edu.es (E.C.-L.); mreyesba@ull.edu.es (M.R.-B.); jpinero@ull.edu.es (J.E.P.); 2 Departamento de Obstetricia y Ginecología, Pediatría, Medicina Preventiva y Salud Pública, Toxicología, Medicina Legal y Forense y Parasitología, Universidad de La Laguna, 38200 San Cristóbal de La Laguna, Spain; 3 Centro de Investigación Biomédica en Red de Enfermedades Infecciosas (CIBERINFEC), Instituto de Salud Carlos III, 28029 Madrid, Spain; 4 Departamento de Bioquímica, Microbiología, Biología Cellular y Genética, Universidad de La Laguna, 38200 San Cristóbal de La Laguna, Spain

**Keywords:** soil, water, *Acanthamoeba*, *Vermamoeba vermiformis*, monitoring, Fuerteventura

## Abstract

Free-living amoebae (FLA) are protozoa ubiquitous in nature, isolated from a variety of environments worldwide. In addition to their natural distribution, some species have been found to be pathogenic to humans. In the present study, FLA presence was evaluated and characterized at the molecular level from different water and soil samples in Fuerteventura Island, Canary Islands, Spain. A total of 31 samples were analyzed by culture and molecular assays (q-PCR and PCR). Moreover, the microbiological quality of the water samples was examined as required by current legislation and international standards. The obtained data revealed that the genus *Acanthamoeba* was the most prevalent genus of FLA in soil samples and the species *Vermamoeba vermiformis* was the most isolated in water samples collected from Fuerteventura by culture and molecular assays, q-PCR, and conventional PCR/Sanger sequencing. On the other hand, a microbiological analysis revealed heterogeneous contamination patterns. *Escherichia coli* was detected in several samples, with some exhibiting high counts while others showed no presence. *Salmonella* spp. appeared in multiple samples, particularly FTVW1, FTVW9, and FTVW13, whereas *Shigella* spp. was only found in one sample (FTVW1). Moreover, q-PCR detection offers advantages such as reduced detection time and cost. In addition, culture was proven to be more effective for confirming FLA viability and isolating a greater variety of FLA. Overall, the occurrence of potentially pathogenic free-living amoebae in habitats related to the human population, as reported in the present study, supports the relevance of FLA as a potential health threat to humans.

## 1. Introduction

Free-living amoebae (FLA) are widely distributed protozoa in the environment that can survive in different habitats such as water, soil, dust and air sources [1,2]. This group is a heterogeneous assemblage of amphizoic amoeboid organisms, which combines several lineages belonging to Amoebozoa and Cercozoa, known to be causative agents of disease in humans and other animals.

Among FLA, some species are causative agents of disease in humans and other animals, such as *Acanthamoeba* spp., *Balamuthia mandrillaris*, *Naegleria fowleri*, *Sappinia diploidea*, *Vermamoeba vermiformis* and *Vahlkampfia* spp. [2,3,4]. Specifically, *Acanthamoeba* spp., *N. fowleri*, *B. mandrillaris*, and *S. diploidea* have been reported as causative agents of encephalitis [3,5,6]. Moreover, other infections are keratitis caused mainly by the genera *Acanthamoeba* and *Vahlkampfia* and less frequently *V. vermiformis* [3,7,8,9,10]. Thus, *V. vermiformis* has been identified as both an etiological agent and a pathogen reservoir and, according to a recent study, it is the cause of a painful ulcer next to the eye [11]. Moreover, *B. mandrillaris* and *Acanthamoeba* spp. cause dermatitis in immunosuppressed individuals [3]. In addition to their pathogenicity, FLA are reservoirs for numerous harmful bacteria and viruses that are clinically significant for both humans and other animals [12]. This has implications for the eradication of bacterial pathogens from water supplies [13].

The Canary Islands constitute an archipelago of eight inhabited major islands and six islets, located in the Atlantic Ocean, between latitudes 27°38′ and 29°25′ North (which is more than 200 km from North to South) and longitudes 13°30′ and 18°19′ West (500 km from East to West, approximately) [14,15]. There have been reports of FLA on five of the eight main Canary Islands [16,17,18,19]. In particular, on one of the eastern islands, Fuerteventura, the greatest variety of free-living amoebae was found in soils and irrigation waters, including the genera *Acanthamoeba* and *Naegleria*, and the species *V. vermiformis*. Considering that Fuerteventura is the second largest island and has many different habitats where amoebae can thrive and pose a health risk to residents and visitors, it underlines the need to monitor their environments regularly. Therefore, the aim of this study was to expand on previous work by including other type samples from different locations and simultaneously evaluate the presence of *Escherichia coli*, *Salmonella* spp. and *Shigella* spp., providing relevant data for the surveillance and control of microbiological risks on the island.

## 2. Materials and Methods

### 2.1. Sampling Site

**Figure 1:**
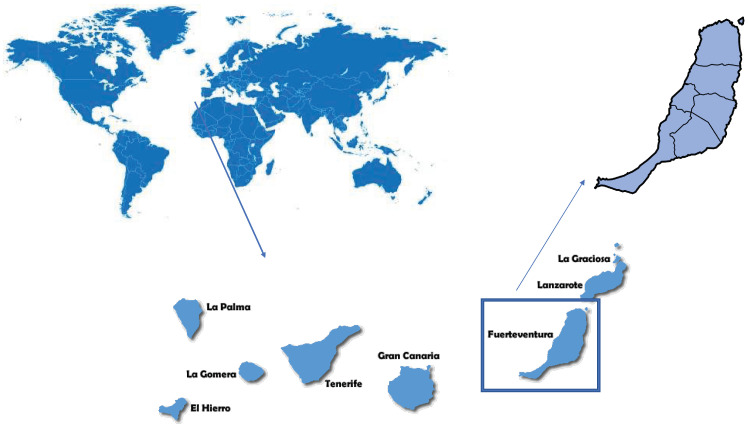
Geographical localization of Fuerteventura Island.

The study was carried out on the volcanic island of Fuerteventura (Canary Islands, Spain), which is located 115 km off the west coast of Africa in the Atlantic Ocean between latitudes 28°45′ and 28°02′ north and longitudes 13°49′ and 14°20′ west (Figure 1). Fuerteventura Island, a UNESCO Biosphere Reserve since 2009, is considered one of the most arid territories in the European Union (mean annual precipitation ≈ 150 mm; evaporation rates ≈ 1800–2000 mm yr^−1^ in evaporimetric tank [20,21,22]. The climatic conditions during the study period, May 2024, were as follows: an average temperature of 18.6 °C, exceeding the historical average by +0.7 °C and in terms of accumulated rainfall, an average of 5.7 mm was recorded, making it a wet month in terms of rainfall [23].

### 2.2. Soil and Water Sampling

In an isolated dry agroecosystem, 31 environmental samples were gathered, 15 from soil and 16 from water sources. Samples from the soil’s surface layer (0–1.5 cm) were collected using sterile 15 mL tubes, and water was collected using 50 mL tubes. The tubes were then kept at 4 °C until processing. To reduce particle dislodgement and turbulence during collection, the tubes were pushed slowly and steadily down the surface. All operations were performed while wearing sterile gloves and shoe covers (Table 1).

### 2.3. Characterization of Bacterial Load of Water

Water samples were diluted to 10^−2^ and 10^−3^, and then subjected to a filtration process using a filtration manifold, through which 100 mL of water was filtered using nitrocellulose membrane filters, 0.45 μm pore diameter (Pall, Madrid, Spain) with the aid of a vacuum pump (Thermofisher, Madrid, Spain). After that, filters were cultured face-up onto selective media culture plates such as *Salmonella*-*Shigella* agar (S/S) and Chromocult agar. The S/S plates were incubated for 48 h at 37 °C, allowing differentiation of *Salmonella* spp., which produced black colonies, and *Shigella* spp., which formed well-rounded, translucent colonies. In contrast, the Chromocult medium was incubated for 24 h at 37 °C, revealing blue colonies of *Escherichia coli* and red-salmon-colored colonies of other coliforms. In all cases, colony-forming units (CFU) were quantified to establish bacterial load in the water samples.

### 2.4. DNA Extraction from Soil and Water Samples

Each soil sample (0.6 g) was dissolved in 1 mL of Page’s Amoeba solution (PAS) and then vortexed for 5 min. After that, 1 mL of the supernatant was added to the Maxwell^®^ RSC (Promega, Madrid, Spain) [24], following the manufacturer’s instructions. Following the process, around 200 μL of pure DNA was collected and stored for q-PCR analysis.

### 2.5. Multiplex Quantitative Real-Time PCR Assay (q-PCR)

A multiplex q-PCR assay was performed for the simultaneous detection of *Acanthamoeba* spp., *Naegleria fowleri*, *Balamuthia mandrillaris*, and *Vermamoeba vermiformis*. Primers and the adapted TaqMan probes used in the multiplex q-PCR assay were adapted in our laboratory from the previously described assay by Qvarnstrom et al. [25] and Córdoba-Lanús et al. [26], and are listed in Table 2.

The q-PCR reactions were performed in a 10 μL final volume, using 10× TaqMan^®^ Multiplex Master Mix (Applied Biosystems, ThermoFisher Scientific), 0.5 μM of each primer, 0.25 μM of the probe and 2 μL of the obtained DNA of each environmental sample. The q-PCR reaction was set up in a QuantStudio 5 real-time PCR machine (ThermoFisher Scientific, Waltham, MA, USA) under the following conditions: a denaturalization step of 3 min at 95 °C, followed by 40 cycles consisting of two steps of 95 °C for 15 s and 60 °C for 1 min. The cycle threshold (Ct) was obtained by the software Design and Analysis v2.4.3 (ThermoFisher Scientific), and the detection limit of the amplicons for the 4 FLA ranged from Ct 34 to 35, based on the previous results obtained by comparison with the DNA standard curves [26]. DNA from axenic cultures of *Acanthamoeba castellanii* Neff (ATCC^®^30011™), *Naegleria fowleri* (ATCC^®^30808™), an environmental isolate H3 of *Balamuthia mandrillaris* [27] and *Vermamoeba vermiformis* (NCBI MT320010) [28] were used as positive controls.

The ParoReal kit *Acanthamoeba* T4 (Ingenetix-GmbH, Vienna, Austria) was used to determine if the *Acanthamoeba* spp. found in the preceding phase match the T4 genotype, which causes more than 86% of *Acanthamoeba* keratitis worldwide. A QuantStudio 3 real-time PCR thermocycler (ThermoFisher Scientific, MA, USA) was used to conduct the q-PCR experiment. This kit uses an internal control with a Cy5 label and a FAM-labeled probe to identify a positive T4 genotype sample [29].

**Table 2:** Primers used in the q-PCR for the detection of the free-living amoebae under study. * Primers sequence described by Qvarnstrom et al., 2006 [25]. ** Forward primer sequence described by Kuiper et al. (2006) [30]. *** This reverse primer sequence was specially designed by Córdoba-Lanús et al. (2024) [26].

Parasite Species	Primers Sequences	DNA Fragment (bp)
*Acanthamoeba* spp. *	AcantF900 (5′-CCC AGA TCG TTT ACC GTG AA-3′) AcantR1100 (5′-TAA ATA TTA ATG CCC CCA ACT ATC C-3′) AcantProb (5′-JUN-CT GCC ACC GAA TAC ATT AGC ATG G-QSY-3′)	180
*Naegleria fowleri* *	NaeglF192 (3′-GTG CTG AAA CCT AGC TAT TGT AAC TCA GT-5′) NaeglR344 (5′-CAC TAG AAA AAG CAA ACC TGA AAG G-3′) NeglProb (5′-VIC-AT AGC AAT ATA TTC AGG GGA GCT GGG C-QSY-3′)	153
*Balamuthia mandrillaris* *	BalaF1451 (5′-TAA CCT GCT AAA TAG TCA TGC CAA T-3′) BalaR1621 (5′-CAA ACT TCC CTC GGC TAA TCA-3′) BalaProb (5′-6FAM-AG TAC TTC TAC CAA TCC AAC CGC CA-QSY-3′)	171
*Vermamoeba vermiformis*	Hv1227F (5′-TTA CGA GGT CAG GAC ACT GT- 3′) ** VermRv (5′ TGCCTCAAACTTCCATTCGC 3′) *** VermProb (5′-ABI-TTG ATT CAG TGG GTG GTG GT-QSY-3′) ***	235

### 2.6. Free-Living Amoebae Isolation, PCR and Molecular Characterization of Isolates

− Water samples: water was centrifuged at 2500 rpm, and the pellet was placed directly on NNA plates with a layer of heat-killed *E. coli*. After that, the plates were incubated at room temperature and observed daily.
− Soil samples: The soil was placed directly into 2% of Non-Nutrient Agar (NNA) plates with a layer of heat-killed *E. coli* and incubated at room temperature, then monitored each day to check the presence of FLA.

Plates with amoebae growth were cloned in NNA plates until a monoxenic culture was achieved, when possible. Page’s morphological key criteria were used to identify positive plates for amoebic growth at the morphological level.

For molecular analysis, DNA from positive samples was extracted from 1 to 2 milliliters of amoebic culture suspension. The plate containing the monoxenic amoeba culture was treated with 4 mL of Page’s Amoeba Solution (PAS) to produce the amoeba suspension. The plate was scraped, the suspension was centrifuged at 2000 rpm for 5 min, and the concentrated amoeba culture was put straight into the Maxwell 16 tissue DNA purification kit sample cartridge (Promega, Madrid, Spain) in accordance with the manufacturer’s instructions and the previously outlined procedure [16]. The NanoDrop Lite Spectrophotometer was used to measure the yield and purity of extracted DNA.

PCR amplification of the 18S rRNA gene from the extracted DNA was performed using specific primers: JDP-1f 5′-GGCCCAGATCGTTTACCGTGAA-3′ and JDP-2r 3′-TCTCACAAGCTGCTAGGGAGTCA-5′ for amoeba presenting morphology corresponding to *Acanthamoeba* spp. [31] and Hv1227f 5′-TTACGAGGTCAGGACACTGT-3′ [30]/VermRV 5′-TGCCTCAAACTTCCATTCGC-3′ [26] for *Vermamoeba vermiformis*. For the family Vahlkampfiidae, we used these primers: VAHL1 5′-GTCTTCGTAGGTGAACCTGC-3′ and VAHL2 3′-CCGCTTACTGATATGCTTAA-5′ [32]. Amplification reactions were performed with a total of 50 μL of mixture, containing 40 ng of DNA, and the PCRs for *Acanthamoeba* spp. and *V. vermiformis* primers were carried out in 35 cycles with denaturation (95 °C, 30 s), annealing (50 °C, 30 s), and primer extension (72 °C, 30 s). Nevertheless, for VAHL primers, amplification reactions were performed in a 50 μL mixture containing 60 ng of DNA, and the PCRs were performed in 35 cycles with denaturation (95 °C, 60 s), annealing (55 °C, 90 s) and primer extension (72 °C, 120 s). A primer extension of 7 min at 72 °C was maintained after the last cycle. Positive PCR products were sequenced using a Macrogen Spain service, and amplification products from all PCRs were examined by electrophoresis using a 2% agarose gel. Several species were identified using sequence homology analysis, which compares DNA sequences from the National Library of Medicine’s (NCBI) Genbank database.

### 2.7. Phylogenetic Analysis

The sequences obtained in this study were aligned using MAFFT v7 with the accurate L-INS-i algorithm [33], and poorly aligned regions were removed with trimAl when necessary [34]. Phylogenetic analyses were conducted with RAxML v8.2.10 [35] under the maximum likelihood framework, employing the GTRGAMMA substitution model for nucleotide alignments. Node support was assessed with 500 bootstrap replicates. The resulting tree was rooted using an outgroup, *Balamuthia mandrillaris*.

## 3. Results

### 3.1. Characterization of the Microbiological Load of Water

Microbiological analysis of water collected at 16 sampling points on the island of Fuerteventura revealed that the presence of *Salmonella* spp. and *Shigella* spp. was sporadic. Significant levels of *Salmonella* were detected in FTVW1, FTVW7, FTVW9, FTVW11, FTVW13 and FTVW14. In contrast, the presence of *Shigella* was less frequent, standing out only in FTVW1, where up to 4000 CFU were counted in the 10^−2^ dilution. Given that ISO 19250:2010 establishes that *Salmonella* should be absent in 250 mL of water in general, both for human consumption and for other sensitive uses [36]. The presence of this bacterium in surface or irrigation water represents a clear indicator of fecal contamination of human or animal origin, and suggests a direct health risk, especially if these waters are used without adequate pretreatment.

The results for coliforms and *Escherichia coli* showed a remarkable variability among samples. Some, such as FTVW1 and FTVW8, FTVW14, showed elevated *E. coli* counts, while others, such as FTVW3, showed no detectable presence of either coliforms or *E. coli*. In general, most of the samples showed low or no counts, with some occasional exceptions of high or even uncountable concentrations, especially in the case of coliforms in sample FTVW8. This uncountable count of these bacteria indicates a significant microbial load in certain areas.

According to ISO 9308-1:2014 [37], which establishes methods for the detection and enumeration of *E. coli* and coliforms in water using the membrane filtration method, it is considered that *E. coli* counts should be less than 1 CFU/100 mL in drinking water, while in recreational or wastewater, the limits may be higher depending on local legislation. In this study, as mentioned above, the *E. coli* values detected in several samples exceeded the recommended limit, indicating microbiological contamination that does not meet the quality standards established by ISO 9308-1.

Overall, the results show a heterogeneous microbiological quality in the analyzed waters, with some locations showing relevant indicators of fecal contamination, while others show low or no levels of pathogenic bacteria and coliforms (Table S1).

### 3.2. FLA Presence Detection by Multiplex q-PCR

The developed q-PCR assays have demonstrated the presence of pathogenic FLA in 83.87% from the evaluated samples (16 water samples and 15 soil samples). Regarding water, 87.5% were positive by q-PCR (14/16). Of these, 43.75% (7/16) tested positive for *Acanthamoeba* spp. and the genotype T4 was positive in all samples for *Acanthamoeba* spp.; 87.50% (14/16) tested positive for *Vermamoeba vermiformis* and 25% (4/16) tested positive for *Balamuthia mandrillaris*. Considering these data, the most prevalent FLA was *V. vermiformis,* which was detected in the 14 samples. Consequently, no sample that was taken contained *Naegleria fowleri* (Table S2).

Regarding soil, 80% were positive by q-PCR (13/15). Of these, 66.67% (10/15) tested positive for *Acanthamoeba* spp., 53.33% (8/15) tested positive for *V. vermiformis* and 33.33% (5/15) tested positive for *B. mandrillaris*. Based on the data, *Acanthamoeba* spp. was the most prevalent FLA being detected in nearly all samples. Furthermore, genotype T4 was the most frequently identified, appearing in 5 out of 10 positive samples (50%). The presence of *N. fowleri* has not been detected in any of the samples (Table S3).

As can be seen in the table, more than one type of amoeba was detected in nine waters and eight soils (Tables S2 and S3). For every FLA found, the q-PCR reaction’s Ct values varied from 23.5 to 34.5 in soil samples and from 22 to 34.5 in water samples. According to Córdoba-Lanús et al.’s (2024) [26] earlier findings, the DNA found in 0.6 g of the examined soil samples would represent 100^−1^ and 10^−1^ amoebae, respectively.

### 3.3. FLA Presence Detection by Culture

From the total of 31 samples, 13 water samples (13/16; 81.25%) and 14 soil samples (14/15; 93.33%) were positive for the presence of FLA in NNA plates After analysis of the 18S rRNA gene (the DF3 region in the case of *Acanthamoeba*), five water samples (5/13; 38.46%) and nine soil samples (9/14; 64.29%) were positive for PCR. For PCR results, 11 amoebae were tested using the specific JDP primers, 2 with *V. vermiformis* primers and 1 with the Vahl primers. The amplicon length varies, at 200 bp for *V. vermiformis* primers and 500 bp for JDP and Vahl primers.

The presence of *V. vermiformis* in the water samples was prominent (10/16; 62.5%). *Acanthamoeba* spp. was the second most frequently found (6/16; 13.04%), with the T4 genotype being the only one detected. *Naegleria pagei*, *Thecamoeba* spp. and *Cercozoa* spp. were isolated in samples of FTVW12, FTVW1, and FTVW8, respectively, with a 6.25% prevalence for each of them in this study (1/16) (Table S4).

In contrast, *Acanthamoeba* spp. were the most abundantly isolated species in soils, with a total of 13 samples (13/15; 86.67%), with the T4 genotype being the most common. The species *V. vermiformis* was isolated in one sample (1/15; 6.67%) (Table S5).

The obtained sequences in the present study have been deposited in the GenBank database under the following accession numbers: PV799991-PV800002. All of them presented ˃95% of homology with the available DNA sequences in this database.

**Figure 2:**
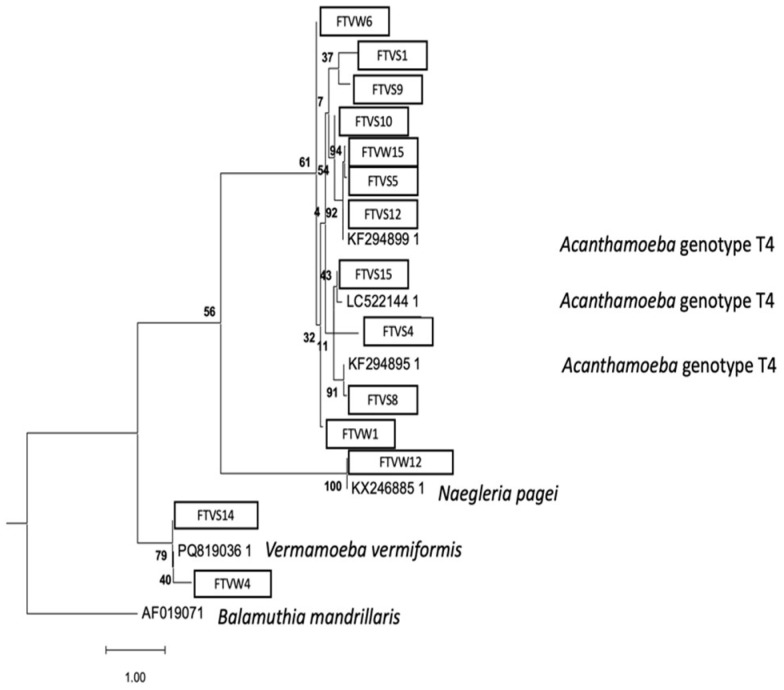
Maximum likelihood phylogenetic tree of the FLA strains obtained from environmental samples based on the 18S rRNA gene analysis, showing the position of each isolate obtained in this study that is marked within boxes. The tree is rooted with *Balamuthia mandrillaris* as the outgroup. The percentage of replicate trees, in which the associated taxa clustered together in the bootstrap test are shown next to the branches (in bold). Scale bar = 1.00 substitutions/site.

The phylogenetic analysis of the 18S rRNA gene is shown in Figure 2:

## 4. Discussion

Fuerteventura is an arid territory with limited water resources [20]. Water quality is essential for both human supply and agricultural use. Therefore, the evaluation of these parasites and bacteria is important. This study confirms the significant presence of FLA and pathogenic bacteria in aquatic and terrestrial environments of Fuerteventura, in agreement with previous research conducted on the island. The high prevalence of FLA, especially of the genera *Acanthamoeba* and *Vermamoeba vermiformis*, is consistent with the findings of Reyes-Batlle et al., who identified these protozoa as the most abundant in soils and waters of the island, as well as in other islands of the Canary Archipelago [28,38]. Nevertheless, the present work considerably expands the spatial and methodological scope in the characterization of FLA and pathogenic bacteria in aquatic and terrestrial environments of the island, since this study incorporates more sensitive molecular tools, such as multiplex q-PCR, which allows the simultaneous detection of several pathogenic species, including *Balamuthia mandrillaris*, absent in previous analyses. Moreover, it broadens the microbiological spectrum by detecting fecal indicators such as *E. coli* and relevant enteric pathogens like *Salmonella* spp. and *Shigella* spp., thus strengthening the sanitary approach. The inclusion of samples not restricted to agricultural environments enables a more representative evaluation of the population’s environmental exposure.

The results obtained in this study revealed a heterogeneous presence of bacteria, indicating fecal contamination. This suggests a possible punctual influence of fecal pollution sources, probably associated with human or livestock activities, deficiencies in sanitation infrastructure, or sporadic meteorological events that favor the transport of organic matter into water bodies [39]. This condition implies a potential risk of enteric pathogen transmission, especially in contexts where water is used without adequate treatment. Conversely, the detection of *Salmonella* and *Shigella* was occasional, although several samples showed significant counts of these bacteria, reinforcing the public health concern. Both species are recognized for causing severe gastrointestinal infections, such as salmonellosis and shigellosis, respectively [40,41,42]. Consequently, their presence in water intended for human consumption, irrigation, or recreation constitutes a direct risk to public health, particularly in an insular environment such as Fuerteventura, characterized by high tourist activity and frequent water exposure in recreational and agricultural settings.

Furthermore, the finding of potentially pathogenic FLA in terrestrial and aquatic environments of Fuerteventura is particularly relevant within the ecological and health context of this island, characterized by extreme aridity and dependence on limited and frequently reused water sources. Abundant presence and diversity were detected through culture and molecular techniques, with *Vermamoeba vermiformis* being the most prevalent species in water and *Acanthamoeba* spp. the most frequent in soil. The detection of the T4 genotype of *Acanthamoeba* spp. is particularly noteworthy, as it is associated with most cases of amoebic keratitis worldwide and granulomatous amoebic encephalitis, and it is considered the main pathogenic genotype for humans [13,43]. Likewise, the identification of *Balamuthia mandrillaris* in both matrices broadens the spectrum of potential risk, since this species has been implicated in fatal cases of granulomatous amoebic encephalitis [44,45].

It is worth noting that these protozoa show remarkable resistance and adaptability to the extreme environmental conditions (low humidity, high solar radiation, and elevated temperatures) that characterize the island. This resilience in arid environments can be partly explained by their resistance to desiccation, their ability to form cysts, and their growth in biofilms present in hydraulic networks and reservoirs [12,13,46]. Indeed, FLA may benefit from rising temperatures and climatic variability associated with global change, thereby expanding its geographic distribution and occurrence in insular ecosystems previously considered low risk [47]. A paradigmatic example of this adaptive capacity is the Atacama Desert (Chile), considered one of the driest places on Earth, where the presence of *Acanthamoeba* has been documented [48].

On the other hand, the integration of molecular tools with conventional microbiological analyses provided a more comprehensive overview of the FLA community present in the studied environments. The high detection rate by q-PCR, even in samples negative by culture, suggests the existence of viable but non-culturable forms, with important implications for health surveillance. Furthermore, q-PCR offers advantages such as reduced detection time and cost [49,50]. Conversely, culture techniques proved effective for confirming FLA viability and detecting a greater variety of species, such as *Naegleria pagei* and *Cercozoa* spp. Among *Naegleria* species, *N. fowleri*, the causative agent of Primary Amoebic Meningoencephalitis (PAM), was not identified in the studied water and soil samples. Hence, PAM cases have not been reported in this island. The *Naegleria* species found in this work, *N. pagei* is present in Europe, North America, Africa, and Asia.

The *Cercozoa* genus is considered a non-pathogenic amoeba; nevertheless, it can contribute to the spread of other pathogenic populations, serving as a potential host for endosymbiotic bacteria [51,52]. Therefore, this reinforces the need to develop broader environmental monitoring programs that include not only classical bacterial indicators but also emerging protozoa such as FLA. This integrated approach is particularly relevant in arid regions with intensive water use, such as Fuerteventura, where water quality directly affects human health, agriculture, and tourism. Regular monitoring allows early detection of health risk situations, prevention of waterborne disease outbreaks, and guidance for corrective actions in sanitation and wastewater management infrastructure. The presence of FLA and pathogenic bacteria in water intended for agricultural or recreational uses represents a potential risk that should be considered in water resource management policies, especially in island regions with structural water limitations. Likewise, further exploration of the relationships between bacteria and amoebae through functional and metagenomic studies is recommended to better understand the ecological dynamics of these microorganisms in arid environments.

## Figures and Tables

**Table 1:** Location of environmental samples (FTV indicates Fuerteventura, W is for water samples and S for soil samples) analyzed for FLA and bacteria detection in Fuerteventura.

	Sample Code	Locality	Coordinates	Samples Type
W A T E R S A M P L E S	FTVW1	Betancuria	28.402567, −14.132785	Creek
	FTVW2	Betancuria	28.403492, −14.131744	Creek
	FTVW3	Betancuria	28.388636, −14.098953	Dam
	FTVW4	Betancuria	28.542434, −14.061964	Ravine
	FTVW5	Puerto del Rosario	28.484910, −13.922606	Ravine
	FTVW6	Pájara	28.260050, −14.162467	Creek
	FTVW7	Pájara	28.254811, −14.176075	Water raft
	FTVW8	Antigua	28.388114, −13.873759	Regenerated water
	FTVW9	Antigua	28.387500, −13.873330	Regenerated water
	FTVW10	Pájara	28.185210, −14.160825	Pond
	FTVW11	Pájara	28.185210, −14.160825	Pond
	FTVW12	Pájara	28.185210, −14.160825	Pond
	FTVW13	Pájara	28.185210, −14.160825	Pond
	FTVW14	Pájara	28.185210, −14.160825	Pond
	FTVW15	Pájara	28.185210, −14.160825	Pond
	FTVW16	Pájara	28.185210, −14.160825	Pond
	FTVS1	Betancuria	28.403492, −14.131744	Ravine
	FTVS2	Betancuria	28.423503, −14.057736	Shore of a creek
S O I L S A M P L E S	FTVS3	Betancuria	28.387584, −14.095719	Dam
	FTVS4	Betancuria	28.509744, −14.030560	Dam
	FTVS5	Betancuria	28.542506, −14.061995	Ravine
	FTVS6	Betancuria	28.543128, −14.061298	Ravine
	FTVS7	Puerto del Rosario	28.484872, −13.922648	Ravine
	FTVS8	Pájara	28.260050, −14.162467	Ravine
	FTVS9	Antigua	28.388114, −13.873759	Regenerated water
	FTVS10	Antigua	28.381031, −13.876235	Regenerated water
	FTVS11	Pájara	28.185027, −14.160198	Edge of Pond
	FTVS12	Pájara	28.185027, −14.160198	Edge of Pond
	FTVS13	Pájara	28.185027, −14.160198	Edge of Pond
	FTVS14	Pájara	28.185027, −14.160198	Edge of Pond
	FTVS15	Pájara	28.185027, −14.160198	Edge of Pond

## Data Availability

The original contributions presented in this study are included in the article/Supplementary Material. Further inquiries can be directed to the corresponding author.

## Supplementary Materials

The following supporting information can be downloaded at: https://www.mdpi.com/article/10.3390/pathogens15010041/s1, Table S1: Escherichia coli and coliform counts in Chromocult medium and Salmonella/Shigella spp. counts (CFU/1000 mL) in S/S medium in the samples analysed; Table S2: FLA species identified by q-PCR in fresh water sources from Fuerteventura. Acanthamoeba spp., Vermamoeba vermiformis, Balamuthia mandrillaris and Naegleria fowleri values (Values are given as the cycle threshold (Ct); Table S3: FLA species identified by q-PCR in soil samples from Fuerteventura. Acanthamoeba spp., Vermamoeba vermiformis, Balamuthia mandrillaris and Naegleria fowleri values (Values are given as the cycle threshold (Ct); Table S4: FLA species isolated from the evaluated water sources in Fuerteventura (NNA: FLA growth in non-nutrient agar culture; PCR: FLA detection by PCR; homology (%) related to NCBI Database sequence); Table S5: FLA species isolated from the evaluated soil samples in Fuerteventura (NNA: FLA growth in non-nutrient agar culture; PCR: FLA detection by PCR; homology (%) related to NCBI Database sequence).
