# Supplementary material for: High Occurrence of Pathogenic Free-Living Amoebae in Arid Environments
Source: Pathogens. 2025 Dec 30;15(1):41. doi: 10.3390/pathogens15010041 (PMC12844910; doi:10.3390/pathogens15010041)
Supplement: Supplementary file 1 [file pathogens-15-00041-s001.zip › pathogens-4055635-supplementary.pdf]

Table S1. *Escherichia coli* and coliform counts in Chromocult medium and *Salmonella*/*Shigella* spp. counts (CFU/1000 mL) in S/S medium in the samples analysed.

	Sample code	Dilution 10^{-2}	Dilution 10^{-3}
S/S	FTVW1	900 CFU *Salmonella* spp.<br>4000 CFU *Shigella* spp.	3000 CFU *Salmonella* spp.<br>9000 CFU *Shigella* spp.
	FTVW2	0 CFU *Salmonella* spp.<br>0 CFU *Shigella* spp.	1000 CFU *Salmonella* spp.<br>0 CFU *Shigella* spp.
	FTVW3	0 CFU *Salmonella* spp.<br>0 CFU *Shigella* spp.	0 CFU *Salmonella* spp.<br>0 CFU *Shigella* spp.
	FTVW4	0 CFU *Salmonella* spp.<br>0 CFU *Shigella* spp.	0 CFU *Salmonella* spp.<br>0 CFU *Shigella* spp.
	FTVW5	0 CFU *Salmonella* spp.<br>0 CFU *Shigella* spp.	0 CFU *Salmonella* spp.<br>0 CFU *Shigella* spp.
	FTVW6	0 CFU *Salmonella* spp.<br>0 CFU *Shigella* spp.	0 CFU *Salmonella* spp.<br>0 CFU *Shigella* spp.
	FTVW7	900 CFU *Salmonella* spp.<br>0 CFU *Shigella* spp.	0 CFU *Salmonella* spp.<br>0 CFU *Shigella* spp.
	FTVW8	200 CFU *Salmonella* spp.<br>0 CFU *Shigella* spp.	0 CFU *Salmonella* spp.<br>0 CFU *Shigella* spp.
	FTVW9	900 CFU *Salmonella* spp.<br>0 CFU *Shigella* spp.	1000 CFU *Salmonella* spp.<br>0 CFU *Shigella* spp.
	FTVW10	100 CFU *Salmonella* spp.<br>0 CFU *Shigella* spp.	0 CFU *Salmonella* spp.<br>0 CFU *Shigella* spp.
	FTVW11	0 CFU *Salmonella* spp.<br>0 CFU *Shigella* spp.	2000 CFU *Salmonella* spp.<br>0 CFU *Shigella* spp.
	FTVW12	100 CFU *Salmonella* spp.<br>0 CFU *Shigella* spp.	0 CFU *Salmonella* spp.<br>0 CFU *Shigella* spp.
	FTVW13	600 CFU *Salmonella* spp.<br>0 CFU *Shigella* spp.	3000 CFU *Salmonella* spp.<br>0 CFU *Shigella* spp.
	FTVW14	100 CFU *Salmonella* spp.<br>0 CFU *Shigella* spp.	5000 CFU *Salmonella* spp.<br>0 CFU *Shigella* spp.
	FTVW15	300 CFU *Salmonella* spp.<br>0 CFU *Shigella* spp.	0 CFU *Salmonella* spp.<br>0 CFU *Shigella* spp.
	FTVW16	0 CFU *Salmonella* spp.<br>0 CFU *Shigella* spp.	0 CFU *Salmonella* spp.<br>0 CFU *Shigella* spp.
	FTVW1	Countless CFU coliforms<br>9600 CFU *E. coli*	Countless CFU coliforms<br>6000 CFU *E. coli*
	FTVW2	Countless CFU coliforms<br>200 CFU *E. coli*	30000 CFU coliforms<br>0 CFU *E. coli*

CHROMCULT	FTVW3	0 CFU coliforms<br>0 CFU *E. coli*	0 CFU coliforms<br>0 CFU *E. coli*
	FTVW4	Countless CFU coliforms<br>0 CFU *E. coli*	Countless CFU coliforms<br>2000 CFU *E. coli*
	FTVW5	Countless CFU coliforms<br>0 CFU *E. coli*	1000 CFU coliforms<br>0 CFU *E. coli*
	FTVW6	Countless CFU coliforms<br>0 CFU *E. coli*	32000 CFU coliforms<br>0 CFU *E. coli*
	FTVW7	Countless CFU coliforms<br>0 CFU *E. coli*	26000 CFU coliforms<br>0 CFU *E. coli*
	FTVW8	Countless CFU coliforms<br>400 CFU *E. coli*	120000 CFU coliforms<br>2000 CFU *E. coli*
	FTVW9	Countless CFU coliforms<br>100 CFU *E. coli*	26000 CFU coliforms<br>0 CFU *E. coli*
	FTVW10	9000 CFU coliforms<br>0 CFU *E. coli*	3000 CFU coliforms<br>0 CFU *E. coli*
	FTVW11	8000 CFU coliforms<br>0 CFU *E. coli*	20000 CFU coliforms<br>0 CFU *E. coli*
	FTVW12	300 CFU coliforms<br>900 CFU *E. coli*	0 CFU coliforms<br>4000 CFU *E. coli*
	FTVW13	Countless CFU coliforms<br>900 CFU *E. coli*	16000 CFU coliforms<br>4000 CFU *E. coli*
	FTVW14	Countless CFU coliforms<br>0 CFU *E. coli*	15000 CFU coliforms<br>0 CFU *E. coli*
	FTVW15	Countless CFU coliforms<br>100 CFU *E. coli*	Countless CFU coliforms<br>0 CFU *E. coli*
	FTVW16	Countless CFU coliforms<br>0 CFU *E. coli*	0 CFU coliforms<br>0 CFU *E. coli*

Table S2. FLA species identified by qPCR in fresh water sources from Fuerteventura. *Acanthamoeba* spp., *Vermamoeba vermiformis, Balamuthia mandrillaris & Naegleria fowleri values (Values are given as the cycle threshold (Ct).*

Water samples	Locality	q-PCR				
		***Acanthamoeba* spp.**	**Genotype T4**	***Vermamoeba vermiformis***	***Balamuthia mandrillaris***	***Naegleria fowleri***
FTVW1	Betancuria	-	-	-	-	-
FTVW2	Betancuria	33	+	34	30.9	-
FTVW3	Betancuria	-	-	33.5	31.8	-
FTVW4	Betancuria	-	-	34.5	-	-
FTVW5	Puerto del Rosario	-	-	33.9	-	-
FTVW6	Pájara	34.4	+	34.5	-	-
FTVW7	Pájara	-	-	34.5	-	-
FTVW8	Antigua	-	-	-	-	-
FTVW9	Antigua	34	+	27.6	-	-
FTVW10	Pájara	-	-	33.2	33.8	-
FTVW11	Pájara	30.2	+	34	-	-
FTVW12	Pájara	-	-	25.3	-	-
FTVW13	Pájara	31	+	24.2	32.8	-
FTVW14	Pájara	22	+	24.5	-	-
FTVW15	Pájara	32.5	+	32.5	-	-
FTVW16	Pájara	-	-	22.3	-	-

Table S3. FLA species identified by qPCR in soil samples from Fuerteventura. *Acanthamoeba* spp., *Vermamoeba vermiformis, Balamuthia mandrillaris & Naegleria fowleri values (Values are given as the cycle threshold (Ct).*

Soil samples	Locality	q-PCR				
		***Acanthamoeba* spp.**	**Genotype T4**	***Vermamoeba vermiformis***	***Balamuthia mandrillaris***	***Naegleria fowleri***
FTVS1	Betancuria	30.9	-	34	35.3	-
FTVS2	Betancuria	26.3	+	29.8	32.1	-
FTVS3	Betancuria	26	+	-	-	-
FTVS4	Betancuria	30	-	-	32	-
FTVS5	Betancuria	-	-	-	31.3	-
FTVS6	Betancuria	26.3	+	34	-	-
FTVS7	Puerto del Rosario	-	-	-	-	-
FTVS8	Pájara	-	-	34	-	-
FTVS9	Antigua	-	-	-	-	-
FTVS10	Antigua	29.4	-	24.5	-	-
FTVS11	Pájara	32.5	+	33.5	-	-
FTVS12	Pájara	34.5	-	-	-	-
FTVS13	Pájara	23.5	+	34.5	34.5	-
FTVS14	Pájara	34	-	30.7	-	-
FTVS15	Pájara	-	-	-	-	-

Table S4. FLA species isolated from the evaluated water sources in Fuerteventura (NNA: FLA growth in non-nutrient agar culture; PCR: FLA detection by PCR; homology (%) related to NCBI Database sequence).

	Sample code	Locality	NNA	PCR	Genus/species	Homology (%)
WATER SAMPLES	FTVW1	Betancuria	+	+	*Acanthamoeba* sp. T4	≥ 95%
		Betancuria	+	-	*Thecamoeba* spp.	
	FTVW2	Betancuria	+	-	*Acanthamoeba* spp.	
		Betancuria	+	-	*Vermamoeba vermiformis*	
	FTVW3	Puerto del Rosario	-	-		
	FTVW4	Pájara	+	+	*Vermamoeba vermiformis*	≥ 95%
	FTVW5	Pájara	-	-		
	FTVW6	Antigua	+	+	*Acanthamoeba* sp. T4	≥ 95%
		Antigua	+	-	*Vermamoeba vermiformis*	
	FTVW7	Pájara	-	-		
	FTVW8	Pájara	+	-	*Cercozoa* spp.	
	FTVW9	Pájara	+	-	*Vermamoeba vermiformis*	
	FTVW10	Pájara	+	-	*Vermamoeba vermiformis*	
	FTVW11	Pájara	+	-	*Acanthamoeba* spp.	
		Pájara	+	-	*Vermamoeba vermiformis*	
	FTVW12	Pájara	+	+	*Naegleria pagei*	≥ 95%
	FTVW13	Betancuria	+	-	*Vermamoeba vermiformis*	
	FTVW14	Betancuria	+	-	*Acanthamoeba* spp.	
		Betancuria	+	-	*Vermamoeba vermiformis*	
	FTVW15	Betancuria	+	+	*Acanthamoeba* sp. T4	≥ 95%
		Puerto del Rosario	+	-	*Vermamoeba vermiformis*	
	FTVW16	Pájara	+	-	*Vermamoeba vermiformis*	

Table S5. FLA species isolated from the evaluated soil samples in Fuerteventura (NNA: FLA growth in non-nutrient agar culture; PCR: FLA detection by PCR; homology (%) related to NCBI Database sequence).

	Sample code	Locality	NNA	PCR	Genus/species	Homology (%)
SOIL SAMPLES	FTVS1	Betancuria	+	+	*Acanthamoeba* sp. T4	≥ 95%
	FTVS2	Betancuria	+	-	*Acanthamoeba* spp.	
	FTVS3	Betancuria	+	-	*Acanthamoeba* spp.	
	FTVS4	Betancuria	+	+	*Acanthamoeba castellani* T4	≥ 95%
	FTVS5	Betancuria	+	+	*Acanthamoeba* sp. T4	≥ 95%
	FTVS6	Betancuria	+	-	*Acanthamoeba* spp.	
	FTVS7	Puerto del Rosario	-	-		
	FTVS8	Pájara	+	+	*Acanthamoeba* sp. T4	≥ 95%
	FTVS9	Antigua	+	+	*Acanthamoeba* sp. T4	≥ 95%
	FTVS10	Antigua	+	+	*Acanthamoeba* sp. T4	≥ 95%
	FTVS11	Pájara	+	-	*Acanthamoeba* spp.	
	FTVS12	Pájara	+	+	*Acanthamoeba* sp. T4	≥ 95%
	FTVS13	Pájara	+	-	*Acanthamoeba* spp.	
	FTVS14	Pájara	+	+	*Vermamoeba vermiformis*	≥ 95%
	FTVS15	Pájara	+	+	*Acanthamoeba* sp. T4	≥ 95%
